# Rationale of using the dual chemokine receptor CCR2/CCR5 inhibitor cenicriviroc for the treatment of COVID-19

**DOI:** 10.1371/journal.ppat.1010547

**Published:** 2022-06-24

**Authors:** Daniel Clark Files, Frank Tacke, Alexandra O’Sullivan, Patrick Dorr, William G. Ferguson, William G. Powderly

**Affiliations:** 1 Department of Internal Medicine, Pulmonary, Critical Care, Allergy and Immunology Section, Wake Forest University School of Medicine, Winston-Salem, North Carolina, United States of America; 2 Medical Department of Hepatology and Gastroenterology, Charité Universitätsmedizin Berlin, Campus Virchow-Klinikum and Campus Charité Mitte, Berlin, Germany; 3 AbbVie Inc., North Chicago, Illinois, United States of America; 4 John T. Milliken Department of Internal Medicine, Division of Infectious Diseases, Washington University School of Medicine in St Louis, St Louis, Missouri, United States of America; Yale University School of Medicine, UNITED STATES

## Abstract

Coronavirus Disease 2019 (COVID-19), caused by Severe Acute Respiratory Syndrome Coronavirus 2 (SARS-CoV-2), has created a global pandemic infecting over 230 million people and costing millions of lives. Therapies to attenuate severe disease are desperately needed. Cenicriviroc (CVC), a C-C chemokine receptor type 5 (CCR5) and C-C chemokine receptor type 2 (CCR2) antagonist, an agent previously studied in advanced clinical trials for patients with HIV or nonalcoholic steatohepatitis (NASH), may have the potential to reduce respiratory and cardiovascular organ failures related to COVID-19. Inhibiting the CCR2 and CCR5 pathways could attenuate or prevent inflammation or fibrosis in both early and late stages of the disease and improve outcomes of COVID-19. Clinical trials using CVC either in addition to standard of care (SoC; e.g., dexamethasone) or in combination with other investigational agents in patients with COVID-19 are currently ongoing. These trials intend to leverage the anti-inflammatory actions of CVC for ameliorating the clinical course of COVID-19 and prevent complications. This article reviews the literature surrounding the CCR2 and CCR5 pathways, their proposed role in COVID-19, and the potential role of CVC to improve outcomes.

## Author summary

This article reviews the literature examining the inflammatory pathways resulting in pulmonary and cardiovascular adverse events associated with the Coronavirus Disease 2019 (COVID-19) and their role in the disease process; it also reviews the mechanism of action and safety profile of cenicriviroc (CVC), a C-C chemokine receptor type 5 (CCR5) and C-C chemokine receptor type 2 (CCR2) antagonist, previously studied in advanced clinical trials for treatment of HIV or nonalcoholic steatohepatitis (NASH), as a possible treatment option to prevent severe respiratory and cardiovascular outcomes in patients with COVID-19.

## Introduction

In late 2019, health authorities first detected an infection caused by a novel coronavirus, Severe Acute Respiratory Syndrome Coronavirus 2 (SARS-CoV-2); this rapidly transmissible virus would go on to create a global pandemic, known as Coronavirus Disease 2019 (COVID-19), infecting over 438 million people and causing over 5.9 million deaths at the time of writing [1]. SARS-CoV-2 is within the same family of betacoronaviruses as Severe Acute Respiratory Syndrome Coronavirus (SARS-CoV) and Middle East Respiratory Syndrome Coronavirus (MERS-CoV) [2]. SARS-CoV-2 infection has a heterogenous clinical presentation, ranging from asymptomatic infection to severe disease and death [2]. Approximately 5% to 10% of individuals develop symptoms of respiratory failure marked by pneumonia and hypoxia [2,3]. This can further develop into acute respiratory distress syndrome (ARDS), multisystem organ failure, and death. Although the initial symptoms and course of disease are a consequence of viral replication, progressive COVID-19 pneumonia appears to be a consequence more of an aberrant immune response to the virus and less due to the viral replication itself [4].

At the time of writing, the US Food and Drug Administration (FDA) had issued approval or emergency use authorization (EUA) for the mRNA vaccines, Comirnaty (BNT162b2; Pfizer-BioNTech), Spikevax (mRNA-1273; Moderna), and the adenoviral vaccine JNJ-7843673 (Janssen) [5]. Other regional and country regulatory authorities have approved additional vaccines (e.g., AZD1222/ChAdOx1 nCov-19, Sputnik-5, and BBIBP-CorV) following successful clinical trials. FDA-approved small-molecule antiviral therapies include Veklury (remdesivir—Gilead Sciences) [6], an IV viral RNA polymerase inhibitor for hospitalized patients; and more recently issued EUA for oral treatments of mild to moderate disease, Paxlovid (nirmatrelvir and ritonavir), for inhibition of the SARS-CoV-2 main protease [7]; and Lagevrio (molnupiravir), for induction of viral RNA error catastrophe [8]. The FDA has also approved or issued EUA for monoclonal antibodies that bind to the spike protein of SARS-CoV-2 to prevent host cell entry. This includes bamlanivimab plus etesevimab, casirivimab and imdevimab, sotrovimab, and bebtelovumab, although activity of these antibodies against emerging dominant variants (especially Omicron) has been reported to be much impaired, with the exception of sotrovimab and bebtelovumab [9–11], to the extent that bamlanivumab use has since been FDA rescinded and bamlanivumab plus etesevimab as well as casirivimab and imdevimab have had their uses limited by the FDA [12]. For preexposure prophylaxis, the monoclonal antibody combinations of tixagevimab plus cilgavimab also have an EUA from the FDA for populations who are immunocompromised and with limited expected response to vaccination [13]. This is important because unvaccinated and immunocompromised patients are at most risk for severe COVID-19 including ARDS [14–18].

Immunomodulators either approved, authorized, or in guidelines for COVID-19 include dexamethasone, a steroidal anti-inflammatory, tocilizumab, an anti-interleukin (IL)-6 receptor monoclonal antibody, and baricitinib, a janus kinase inhibitor [19–22]. Although the list of agents is growing, efficacy remains limited, and further agents to improve outcomes by targeting the pathological mechanisms of COVID-19 are desperately needed. In addition to new drug therapies in development, existing drug or clinical development candidates are also being examined for their potential role in SARS-CoV-2 treatment, including antiviral agents and immunomodulators. Identifying potential targets that mediate the immune response to regulate the respiratory and vascular sequelae could have a profound impact in reducing the severity of disease in SARS-CoV-2 patients; in severe disease, respiratory failure is universally present and may result from excessive cytokine (including chemokine) release from activated immune cells [23,24]. One such drug therapy that can modulate cytokine activity is cenicriviroc (CVC), a C-C chemokine receptor type 5 (CCR5) and C-C chemokine receptor type 2 (CCR2) antagonist, previously studied in clinical trials for consequential antiviral activity against HIV-1 due to inhibiting the CCR5-mediated HIV entry into T cells [25,26]. CVC has also been examined in clinical trials for the treatment of nonalcoholic steatohepatitis (NASH), in which inflammation and hepatocyte injury occur leading to liver fibrosis [27–29]. As inflammation and cytokine (including chemokine) release can occur via similar receptor pathways in pulmonary injury and SARS-CoV-2, we reviewed the literature surrounding the CCR5 and CCR2 pathways and the rationale for CVC as a potential agent in the treatment of patients with COVID-19 [26,30]. COVID-19 may be characterized by pathologies induced by the separate waves of infection caused by distinct variants, including those seen for the Omicron variant that became predominant at an alarming rate across many geographic areas due to its relative increased infectivity. Early research indicates this variant may be associated with reduced disease severity and ARDS, although significant virulence is still apparent [31,32], indicating current interventional mechanisms to reduce disease severity are likely to still be valid for many. Unvaccinated populations are overwhelmingly the most affected by disease severity and ARDS, as are those with compromised immunity and limited immune response to vaccine and infection (e.g., cancer patients), [33,34] making therapeutic interventions particularly important for this population, even in light of likely reduced disease severity associated with the Omicron variant. Therapeutic interventions may become increasingly important due to reduced vaccine-mediated immune susceptibility (especially humoral) of this variant in light of extensive epitope changes [35,36]. CCR2 and CCR5 are widely reported to enable trafficking and signaling of immune-dampening myeloid suppressor cells [37–39] and reduce vaccine immune responses [40–43]. Considering this and aberrant myeloid trafficking, including myeloid-derived suppressor cells being a signature of COVID-19 [44,45], CVC use as a vaccine adjuvant may have some specific merit for further research.

## Chemokines and their coreceptors: CCR2 and CCR5

Chemokines, a family of cytokine leukocyte chemoattractants, are a group of immunoregulatory mediators that can direct leukocyte infiltration, positioning, and activation by acting at specific receptors [2]. Chemokines play an important role in trafficking cells during an immune response [46]. During a respiratory virus infection, inflammatory cytokines and chemokines are induced. Inflammatory cells and leukocytes are recruited into local tissue; in SARS-CoV, elevated cytokine and chemokine expression are found in SARS-CoV–infected cells [47].

**Fig 1:**
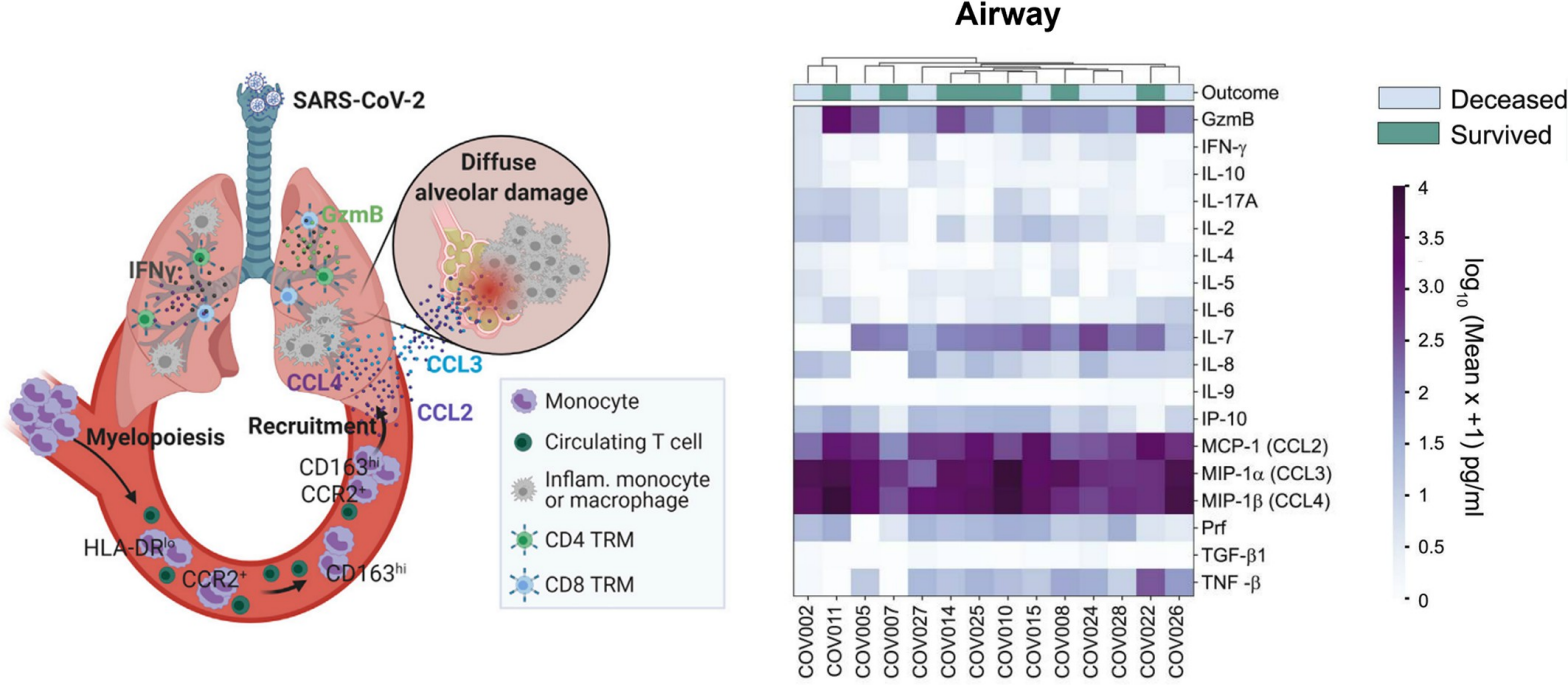
CCR2 mediated recruitment of aberrant myeloid compartment and high CCR2 and CCR5 ligands. Left: Diagrammatic summary representation of CCR2- and CCR5-mediated recruitment of aberrant myeloid compartment via elevated airway CCR2 and CCR5 agonist ligand expression in airways of patients with severe COVID-19 infection. Right: Heatmap showing inflammatory mediators in airway samples from 14 COVID-19 patients (*x* axis) highlighting specifically elevated CCR2 and CCR5 cognate ligands MCP-1 [CCL2], MIP-1α [CCL3], and MIP-1β [CCL4] levels (average elevation relative to uninfected controls graded in purple as per key). Reprinted from *Immunity*, 54 (4), Szabo PA, et al. Longitudinal profiling of respiratory and systemic immune responses reveals myeloid cell-driven lung inflammation in severe COVID-19. 797–814. Copyright (2021), with permission from Elsevier et al. CCL, chemokine-chemokine ligand; CCR, chemokine-chemokine receptor; COVID-19, Coronavirus Disease 2019; MCP, monocyte chemoattractant protein; MIP, monocyte inflammatory protein.

One such chemokine is C-C chemokine ligand 2 (CCL2), a potent cognate agonist of CCR2 [48]. Monocytes, macrophages, vascular endothelial cells, fibroblasts, and smooth muscle cells can secrete CCL2 [49]. In turn, monocytes are the main leukocyte population expressing CCR2 that are being recruited to sites of inflammation via CCL2 [50]. CCL2 thus induces a positive feedback loop by promoting inflammation, thereby releasing additional inflammatory mediators [49,51]. CCL2 is associated with several inflammatory disorders of the lung, including ARDS, the syndrome associated with severe COVID-19, as well as adverse cardiovascular outcomes (**Fig 1**) [47,52–56].

CCR5 and its cognate agonist ligands (CCL3, CCL4, CCR3L1, and CCL5) have long been associated with airway inflammation in both allergic and infectious settings [57]. CCR5 is expressed on several cell types, including T cells, macrophages, vascular cells, and dendritic cells [58,59]. Last, CCR5 is a coreceptor for HIV cell entry (with CD4 being the primary receptor), which underpins its efficacy in HIV infection.

## Association of CCR2 and CCR5 in adverse sequelae associated in COVID-19

### Pulmonary sequelae

**Fig 2:**
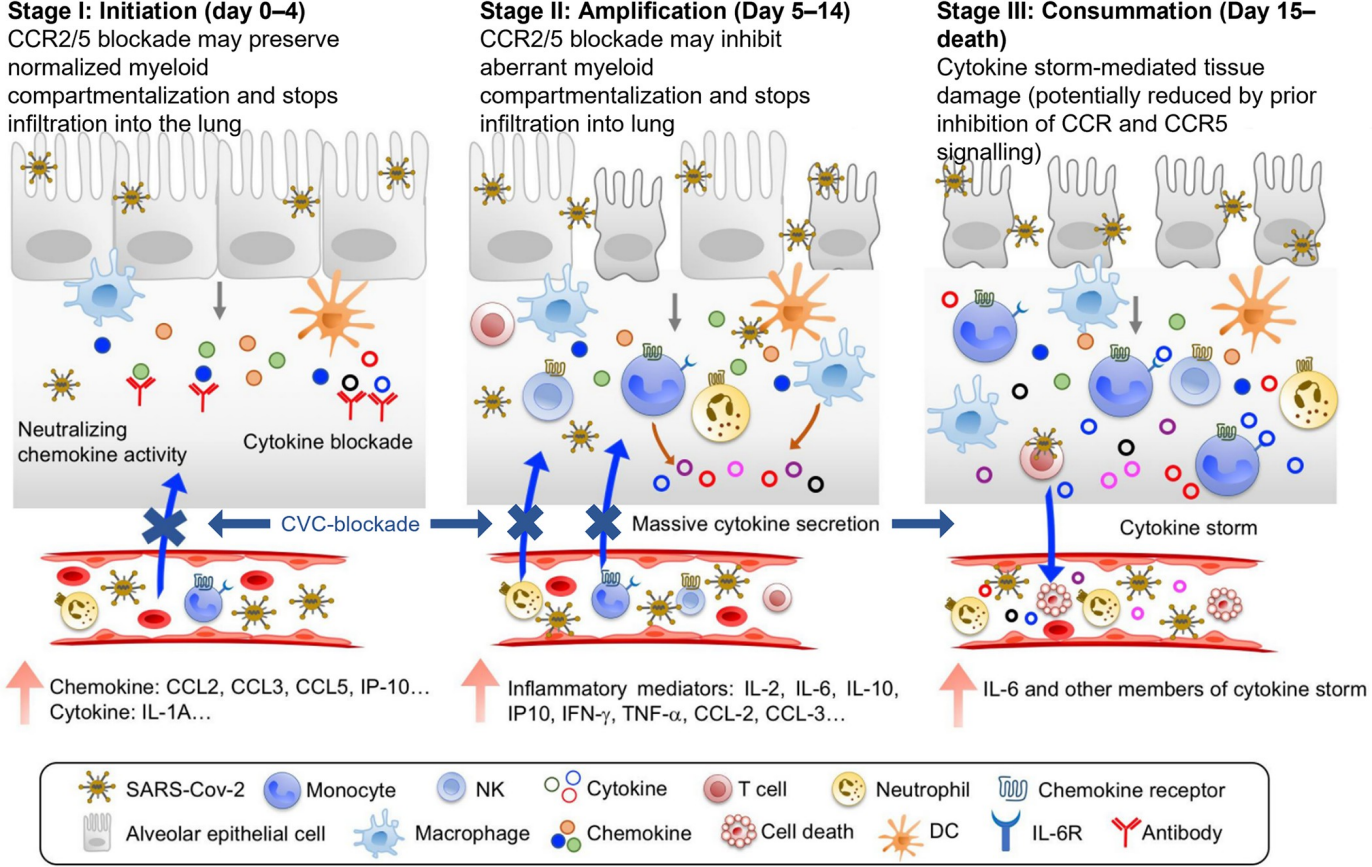
Three stages of immunological pathway leading to mortality in COVID-19: Stage I (Initiation), with early induction of predominant chemokines upon SARS-CoV-2 infection and viral sepsis. Treatment at this stage with CVC (highlighted in blue text and arrows) is postulated to maintain normalized myeloid compartmentalization at early stage of infection, or block aberrant myeloid infiltration upon CCL2, 3, and 5 signaling following SARS-CoV-2 infection and consequent cytokine amplification (Stage II), and subsequent tissue damage and eventual death (Stage III—consummation). Lu L, et al. Preventing Mortality in COVID-19 Patients: Which Cytokine to Target in a Raging Storm? *Front Cell Dev Biol*. 2020;8:677. https://doi.org/10.3389/fcell.2020.00677. CCL, chemokine-chemokine ligand; CCR, chemokine-chemokine receptor; COVID-19, Coronavirus Disease 2019; CVC, cenicriviroc; IL, interleukin; IP, interferon gamma-induced protein; SARS-CoV-2, Severe Acute Respiratory Syndrome Coronavirus 2; TNF, tumor necrosis factor.

Multiple studies have reviewed the roles of CCR2 and CCR5 in mediating respiratory and vascular sequelae across various diseases including COVID-19. Lung injury in ARDS associated with COVID-19 may be associated with dysregulation of inflammatory cytokines, similar to SARS-CoV [60,61]. The Genetics of Mortality in Critical Care (GEnOMICC) genome-wide association study evaluated 2,244 critically ill patients with COVID-19 from 208 United Kingdom intensive care units where high expression of CCR2 was found to be associated with severe COVID-19 via transcriptome-wide association in lung tissue [62]. In SARS-CoV–infected mouse model studies, enhanced production of tumor necrosis factor (TNF) α, IL-6, CCL2, chemokine-chemokine ligand 5 (CCL5), and other chemokines were observed and correlated with the lung migration of macrophages and plasmacytoid dendritic cells [63]. Enhanced cytokine production observed in the lungs including CCL2 and CCL5 along with pneumonitis observed by day 7 [63]. CCL2 is up-regulated early in the stages of acute infection. As the disease progresses, both CCL2 and CCL5 are up-regulated. A similar breakdown of the infection and cytokine elevation longitudinal patterns in humans with SARS CoV-2 infection was reported by Lu and colleagues [64], which proposed 3 stages of infection and cytokine-mediated sequalae, of which the CCR2 and CCR5 cognate agonistic ligands played a significant part. Based on these findings, inhibiting the CCR2 and CCR5 pathways could benefit patients in both early and late stages of infection; optimal administration of CVC during several phases of the SARS-CoV-2 infection may attenuate or prevent inflammatory consequences of COVID-19 and prove beneficial by avoiding excessive monocyte recruitment (**Fig 2**) [63,64].

As the SARS-CoV-2–infected epithelial cells and macrophages in the airways express high levels of CCL5, one pilot study including 10 patients evaluated leronlimab (Pro-140), a monoclonal antibody CCR5 inhibitor, to disrupt the CCL5-CCR5 axis associated with the immune cell infiltration and associated cytokine storm and subsequently the pulmonary sequelae caused by pro-inflammatory leukocytes [65,66]. Although initial data indicated a possible association between CCR5 blockade and reduction in disease markers in uncontrolled studies, more comprehensive clinical trials have failed to demonstrate a clear medical benefit with this antibody in patients with severe disease [65,67]. Localized vasoconstricor responses to elevated chemokine–chemokine ligand 4 (CCL4) via CCR5 agonism may be important in also restricting respiratory function due to SAR-CoV-2 infection, and blockade of this by CCR5 antagonists has been demonstrated [68]. Leronlimab and maraviroc are monoclonal antibody and small-molecule ligands of CCR5, respectively, which are approved drugs for the treatment of HIV infection and operate by blocking CCR5-tropic HIV entry into CD4+ host cells [69,70]. Maraviroc is being investigated for efficacy in various COVID-19 clinical studies (hospitalized patients; see NCT04441385, NCT04475991, NCT04435522, and NCT04710199) by virtue of being a functional antagonist of the CCR5 receptor, postulated to disrupt the associated cognate chemokine-mediated adverse immune-pathophysiology of infection, as discussed later in this manuscript. Results from these studies are awaited. Leronlimab has also been studied in COVID-19 clinical trials, but the largely unfavorable efficacy outcomes in studies may be a consequence of leronlimab being a potent CCR5 ligand and HIV entry inhibitor, but with limited functional antagonistic properties against cognate ligand signaling [71–73]. Like maraviroc, CVC is a potent small-molecule inhibitor of HIV entry/replication on the basis of high affinity binding to CCR5, to block HIV gp120 binding, but also cognate ligand (chemokine) binding and functional signaling [74]. However, unlike maraviroc, CVC possesses additional potent functional antagonistic properties against CCR2 [75] to block additional immune-pharmacologies that are implicated in COVID-19 disease pathology, as detailed in this manuscript. Indeed, CCR5 and CCR2 have interrelated pharmacologies in immune signaling, particularly for the inhibitory myeloid compartment, which may overcome potential interreceptor redundancy or enable a synergistic effect in limiting COVID-19 adverse immunopathology [76–78]. Given this, investigations into CVC as a dual CCR2/5 functional antagonist offer a unique rationale for the potential treatment of COVID-19.

### Cardiovascular sequelae

In addition to respiratory involvement, patients afflicted with COVID-19 often experience cardiovascular complications or have an exacerbation of underlying cardiac disease [79]. While the exact mechanism of this has not been fully elucidated, current evidence points toward an inflammatory response underpinning the adverse cardiovascular outcomes in COVID-19 patients. In a cross-sectional cohort study of 130 patients (ranging in severity of COVID-19) where peripheral blood mononuclear cells were analyzed, surface proteome, T/B lymphocyte antigen receptors, and single-cell transcriptome analyses revealed nonclassical monocytes were largely expanded and expressed complement transcripts (CD16 + C1QA/B/C+) that sequestered platelets and were predicted to replenish the alveolar macrophage pool in COVID-19 [80]. Other research, including in vitro and in vivo studies, has shown that biomarkers of inflammation such as IL-6, IL-8, C-reactive protein, and CCL2 are associated with thrombus development along with leukocytes [81].

Chemokines such as CCL2 and CCL5, along with their coreceptors CCR2 and CCR5, respectively, have long been associated with vascular disease [78,82,83]. CCL2/CCR2 and CCL5/CCR5 recruit monocytes to migrate to sites of inflammation including atherosclerotic plaques; circulating monocytes can trigger tissue factor expression via the release of cytokines from activated platelets and endothelial cells. A signature marker of COVID-19–associated coagulopathy is the presence of neutrophil extracellular traps, which recruit myeloid cells to the culprit site via CCL2 [84–86]. Early recruitment of neutrophil and monocytes trigger factor XII-dependent coagulation and tissue factor delivery thus contributing to the formation of a thrombus [87]. In one study, deep vein thrombosis was induced in wild-type and growth-arrest–specific 6 (Gas6)-deficient mice [81]. Gas6 promoted the recruitment of inflammatory monocytes via CCL2/CCR2. Inflammatory monocyte recruitment via these pathways also occurs in other cardiovascular diseases such as myocardial infarction [49]. A study, examining the expressions of CCL2 and CCR2 in the plasma, found that in 80 STEMI (ST-Elevation Myocardial Infarction) patients with platelet high response, patients had higher expressions of CCL2 or CCR2 than those patients with a platelet normal response. Exogenous recombinant human CCL2 increased platelet aggregation and granule secretions in vitro; these were abolished by a CCR2 inhibitor or a CCL2 neutralizing antibody [49]. These studies further support the theory that the CCL2/CCR2 pathway is vital in cardiovascular events. Given the adverse cardiovascular-related events seen in COVID-19, blockade of CCR2 could potentially reduce these adverse outcomes by decreasing the amount of circulating and infection site accumulation of inflammatory monocytes and other myeloid populations [79,81,88,89].

## Mechanism of action of CVC

CVC is an orally bioavailable, small-molecule chemokine receptor antagonist with similar in vitro potency against cognate ligand binding to both CCR2 and CCR5 receptors (IC50 = 2 to 6 nM) [26,74]. It is an immunomodulator that can decrease the transmigration of immune cells through the blockade of CCR2 and CCR5, thereby preventing monocytes (that differentiate into inflammatory tissue macrophages) and lymphocytes from penetrating lung tissue [90]. The mechanisms of monocyte recruitment to injured lungs and their contribution to inflammatory macrophages appear to be very conserved across tissues, because similar (monocyte-derived) macrophage tissue phenotypes can be observed and mediate inflammation in models of lung injury, as well as, for example, liver injury [91–93].

CVC was initially developed as an anti-HIV drug by Takeda and then Tobira, prior to acquisition by Allergan for investigation in liver disease, namely liver fibrosis with NASH (Allergan subsequently acquired by AbbVie). CVC displays potent, selective anti–HIV-1 activity via binding to CCR5 as a coreceptor of HIV-1 and to prevent virus entry into the cell [25,26]. CVC is efficacious in treating HIV infection with HIV-suppressive activity at doses associated with a highly favorable safety profile as demonstrated in a comprehensive Phase 2b clinical study compared with the then standard of care (SoC) comparator efavirenz [25]. High-level dual receptor blockade was demonstrated as highlighted by the high levels of viral suppression (not achievable without 100% CCR5 occupancy) and dose-dependent increases of CCL2 [25,94]. Elevation of CD4 count was numerically greater in the CVC arms than SoC comparator arms (no statistical analysis reported), and the myeloid inflammatory marker sCD14 was reduced in the CVC arms, but elevated in the SoC arms, with this difference being statistically significant [25]. Despite this encouraging profile as a direct-acting antiviral candidate agent for HIV infection, it was not further developed as an HIV clinical candidate following its acquisition by Allergan, but investigated instead for potential therapy of NASH with liver fibrosis, due to the pharmacologies associates with CCR2 and CCR5 in this disease [27,28]. In a Phase 2b study, CVC showed an improvement in liver fibrosis compared to placebo after 1 year of therapy with similar safety profiles between both CVC and placebo groups. It was also associated with reduced levels of markers of cardiovascular outcomes such as C-reactive protein and fibrinogen and biomarkers of inflammation such as IL-6 and IL-1β [27,28]. Despite mechanistically associated evidence of efficacy, the AURORA Phase 3 study was terminated early due to lack of efficacy based on the results of the planned interim analysis of Part 1 data. While no efficacy data for CVC in animal models of SARS-CoV-2 infection exist, there is evidence from preclinical in vivo models (in mice) that CCR2/CCR5 inhibition by means of CVC administration suppresses the inflammatory-mediated organ injury [95–97]. In models of acute liver injury to mice (by acetaminophen or the hepatotoxin carbon tetrachloride), CCR2 specifically inhibits the recruitment of monocytes into injured liver that give rise to inflammatory monocyte-derived tissue macrophages [96]. Of note, the number of Kupffer cells in the liver (tissue-residing macrophages), remains unaltered upon CVC administration, suggesting that only freshly recruited inflammatory cells are blocked, with preservation of basic homeostatic functions of tissue phagocytes (such as defense against bacteria or other infectious threats). In longer-term injury preclinical in vivo models reflecting NASH, treatment with CVC prevents fibrosis in the liver [95].

These same receptors that play a role in inflammation in hepatic injury may also play a vital role in the immune response that occurs in patients with moderate and severe COVID-19 infections. In one study, CVC was studied for its inhibitory effect on the replication of SARS-CoV-2 in cell cultures [26]. CVC was found to be a selective but fairly weak inhibitor of the viral replication (IC50 for virus-induced cell destruction and viral RNA levels were 19.0 and 2.9 μM, respectively). Such low levels of potency may not be sufficient for a direct antiviral effect, but the potent blockade of key immune populations, such as myeloid-derived suppressor cells may increase the effector B and T cell lymphoid populations for indirect immune-based antiviral activity as seen in other viral infections [37,98–100]. We therefore predict that each of the CCR2 and CCR5 receptors has a complementary role in infection-associated inflammation and tissue sequelae in COVID-19 with CCR2/CCL2 seen in both early and late stages of infection and CCR5/CCL5 seen later in the infectious process [63]. Blockade of both may also disable inter-receptor compensatory mechanisms of these 2 closely related G-protein coupled receptors (GPCRs): CCR2 and CCR5 are key mediators of myeloid cell trafficking and migration into tissues and lymphoid regulation. By blocking the CCR2 and the CCR5 pathways, it is anticipated that the administration of CVC may be beneficial in potentially preventing or reversing the pulmonary and vascular sequelae associated with COVID-19.

## Safety of CVC

CVC is a well-tolerated oral formulation with most adverse events considered mild or moderate; the most common side effects reported are nausea, headache, and diarrhea [25,28]. CVC should be administered with food for optimal absorption. There were no major safety signals in over 2,000 patients exposed to CVC, including vulnerable patient populations such as patients with HIV-1 or patients with liver cirrhosis in CVC clinical trials [25,27,28,101]. CVC does not have apparent dose or exposure-related safety signals, and there is no evidence of promoting (bacterial) infections, including in HIV–positive patients. In preclinical models, CVC inhibited functioning of monocytes and macrophages but other immune cell populations such as neutrophils or lymphoid cells were not adversely affected [102]. Functional blockade of CCR5 by maraviroc and CCR2 by clinical candidate agents to date has met with a fairly benign safety profile in patients across a range of indications. The homozygous Δ32 mutation of CCR5 has been reported as less prevalent in COVID-19 patients, with transcript levels higher in patients versus controls [103,104], although disease course has been reported with no association [105]. However, of particular note is the strong association of the Δ32 CCR5 genotype with increased susceptibility to West Nile virus infection [106,107]. In areas of high West Nile Virus prevalence, the potential utility of maraviroc or CVC for the treatment or prophylaxis of COVID-19 would need significant consideration. A higher dose than what has been used in NASH could potentially be advantageous in COVID-19 patients, as this might ensure faster CCR2 and CCR5 inhibition (i.e., target engagement) of CVC. There is potential for drug-drug interactions with strong cytochrome P450 (CYP 450) 3A4 inhibitors; while remdesivir is a substrate and inhibitor of CYP3A4 in vitro, the clinical relevance of these in vitro findings has not been established [108]. To this end, although CVC as a direct-acting anti-HIV agent was no longer pursued following its acquisition from Tobira by Allergan, and the limited efficacy observed in Phase 3 for NASH with liver fibrosis during its tenure with Allergan and now AbbVie, the pharmacologies and safety profile of this clinical candidate made a case for its evaluation in COVID-19.

## Conclusions

The worldwide spread of SARS-CoV-2 and the associated morbidity and mortality have led to an urgent need for additional therapies to mitigate, including pulmonary and vascular complications of COVID-19. This review describes the role of the CCL2/CCR2 and CCL5/CCR5 chemokine pathways associated with amplification of inflammatory responses in COVID-19 and the role of CVC in inhibiting this pathway [109]. CCL2/CCR2 are critical for monocyte and macrophage migration, a potential mechanism may be monocyte infiltration into the lungs via airway specific expression of CCL2/CCR2 in patients with severe COVID-19 [3,47]. CCL2 contributes to monocyte recruitment in acute lung injury (and subsequent neutrophil-mediated tissue injury) as demonstrated in multiple animal studies [2,63]. CCL2 is up-regulated into the lungs of patients with ARDS, which then induces the migration of circulating CCR2 positive inflammatory cells into the alveoli; airways of patients with COVID-19 express pro-inflammatory mediators, including CCL2; airway myeloid cells propagating inflammatory responses in COVID-19 is further supported by the excessive CCL2 levels found in airways, but not blood in COVID-19 patients versus healthy controls [3,110]. Targeting airway-derived cytokines, such as CCL2, via a CCR2 antagonist may be effective in reducing lung damage and preventing further respiratory sequelae in severe COVID-19 [3]. CCL5 was also expressed >100-fold in SARS-CoV patients with a return to baseline of inflammatory markers such as IL-6 with the administration of leronlimab, further supporting that both CCR2 and CCR5 receptors play a role in the inflammatory airway processes [65]. Cardiovascular studies have demonstrated higher expression of CCL2/CCR2 increased the risk for higher platelet response, atherosclerosis, and thrombus formation [49,81,88]. CCR2 and CCR5 may be potential targets for inhibiting airway and cardiovascular inflammatory processes and reducing lung and cardiovascular damage in those inflicted with SARS-CoV-2 [3].

CVC, a dual, potent CCR2 and CCR5 inhibitor, has demonstrated its effect on mitigating inflammatory pathways in both HIV-1 patients and patients with NASH along with decreasing HIV-1 RNA [25,27,28]. It is theorized that CVC could potentially have a similar effect with respect to reducing adverse inflammatory effects associated with COVID-19. By inhibiting both the CCR2 and CCR5 receptors, CVC may decrease the migration of circulating immune cells early in the infectious process as well as inhibiting tissue-based immune cells at later stages, with subsequent effects of decreasing both pulmonary and vascular sequelae associated with the increased of inflammatory markers. Cell culture studies have demonstrated that CVC is a modest inhibitor of SARS-CoV-2 in vitro, although indirect antiviral activity may be more likely a consequence of CVC-dependent block of immunosuppressor cell infiltration to infection sites, such as CCR2- and CCR5-dependent myeloid suppressor cells [26,38,99,100]. CVC has been studied at doses of 100 mg, 150 mg, and 200 mg and found to be well tolerated with most adverse events mild to moderate in severity [25,27,28].

Further research is needed to determine the utility of CVC in treating patients with moderate to severe COVID-19. At the time of publication drafting, there are currently 3 ongoing studies of CVC in COVID-19 patients: I-SPY/COVID Clinical Trial (NCT04488081), ACTIV-1/NIAD/NIH Consortium Study (NCT04593940), and the single-center Charité trial of CVC treatment for COVID-19 patients in Germany (NCT04500418) [111–113]. The I-SPY trial discontinued testing of CVC as it met the predefined futility criterion, defined as at least 90% probability that the hazard ratio for time to recovery is less than 1.5 compared with the control arm [114]. It should be noted that dexamethasone was included in the treatment and participants in this study were critically ill. The other trials include less severe infection in hospitalized COVID-19 patients. Given the benign safety profile of CVC, the oral bioavailability, and the multimodal pharmacologies that align with disrupting COVID-19 pathology, investigating CVC in early infection, mild disease, and in the post-acute COVID-19 populations also have merit.
